# Sustainable multifaceted HPLC approach for concurrent quantitation of an octa-mixture used in upper respiratory therapy with a five-dimensional sustainability assessment

**DOI:** 10.1038/s41598-026-45971-7

**Published:** 2026-04-13

**Authors:** Hadeel A. Khalil, Maram G. Hafez, Feda A. H. Elgammal, Tarek S. Belal, Dina A. Gawad

**Affiliations:** 1 Pharmaceutical Analytical Chemistry Department, Faculty of Pharmacy, University of Alexandria, Elmessalah, Alexandria, 21521 Egypt; 2 Faculty of Pharmacy, University of Alexandria, Elmessalah, Alexandria, 21521 Egypt

**Keywords:** Erdosteine, Paracetamol, Amoxicillin, Albuterol, Guaifenesin, Chlorpheniramine, Parabens, HPLC, Multianalyte method, Sustainable chemistry, Chemistry, Drug discovery

## Abstract

Upper respiratory tract disorders are seriously prevalent disorders that impact people daily all over the world. Antipyretics, analgesics, bronchodilators, antitussives, expectorants, mucolytics, antihistamines, and antibiotics are among the pharmacological classes that are often administered either alone or in combination to treat and manage the symptoms attributed to upper respiratory tract disorders. In the rising era of green analytical chemistry, the development of broad, multi-analyte analytical techniques that can separate and quantify numerous pharmacologically related substances simultaneously is becoming of increasing significance. This work introduces a new, concise, accurate, and robust high performance liquid chromatography method with diode array detection approach for the simultaneous assay of eight substances, namely; Albuterol, Erdosteine, Paracetamol, Amoxicillin, Chlorpheniramine, Guaifenesin and two of the most frequently encountered preservatives that are incorporated in upper respiratory tract medications; Methyl and Propyl Parabens in their bulk and pharmaceutical formulations. An Inertsil octadecyl silane-3 (4.6 × 250 mm, 5 μm) column and a gradient mobile phase system consisting of methanol and 0.025 M potassium dihydrogen orthophosphate buffer (pH 4) were employed to achieve the separation. Chromatograms were detected at 225 nm for Albuterol and Chlorpheniramine, 230 nm for Guaifenesin and Amoxicillin, 236 nm for Erdosteine, 250 nm for Paracetamol, and 256 nm for Methyl and Propyl Parabens using the diode array detector. The suggested approach was successfully verified in compliance with the International Council for Harmonization guidelines with low values of percentage relative error and percentage relative standard deviation (< 2%) and strong correlation coefficients (> 0.9991), demonstrating its good accuracy, precision and linearity. Additionally, an in-depth five-sided examination was conducted to demonstrate the suggested method’s greenness, blueness, violet innovation and sustainability profiles using the analytical GREEnness metric, analytical eco-scale, blue applicability grade index, click analytical chemistry index, violet innovation grade index and white analytical chemistry assessment metrics. This work represents the establishment of a broad spectrum, multianalyte, versatile methodology that would provide an indispensable asset for quality control laboratories in terms of its affordability, speed, sensitivity, and sustainability.

**Supplementary Information:**

The online version contains supplementary material available at 10.1038/s41598-026-45971-7.

## Introduction

Upper respiratory tract disorders (URTDs) are acute infections that are brought about by a variety of viruses and bacteria^1^. These contribute to a number of illnesses, including acute bronchitis, common cold, allergic rhinitis, sinusitis, influenza, and respiratory distress syndromes^1^. URTDs are considered as highly prevalent conditions that daily affect populations globally. In fact, over forty million workdays and school days are missed as a result of upper respiratory tract illnesses, which reflects as an immense financial burden^2,3^.

URTDs are commonly associated with numerous symptoms as nasal discharge, congestion, sore throat, fever, cough, sinus pain, malaise, and watery eyes^4^. Normally, URTDs symptoms can be controlled using over-the-counter (OTC) drugs, however, in some cases the use of prescribed medications is essential for resolving these complications. Treatment and management of the symptoms associated with URTDs necessitates the administration of several pharmacological classes including antipyretics, analgesics, bronchodilators, antitussives, expectorants, mucolytics, antihistamines, and antibiotics which can be taken individually or in combination^5^.

**Fig. 1:**
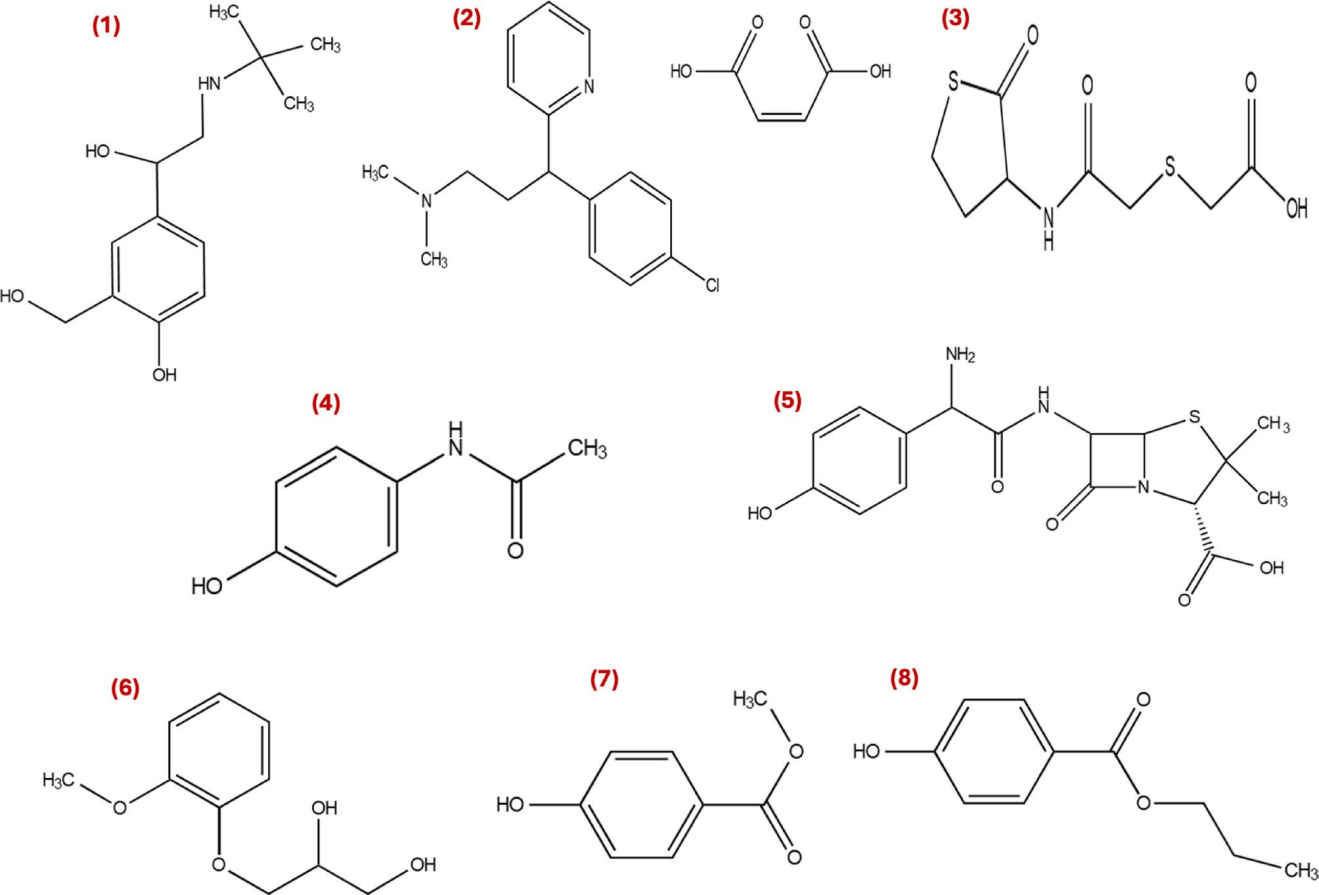
Chemical Structures of (1) ALB, (2) CHR maleate, (3) ERD, (4) PAR, (5) AMX, (6) GUA, (7) MPB, (8) PPB.

This work attempts to develop an ecofriendly multianalyte HPLC-DAD platform for the quantitation of some compounds utilized in the management of URTDs in bulk and their pharmaceutical formulations. Representative examples of the different pharmacological classes were investigated to develop a single analytical method capable of quantitating multiple analytes with acceptable accuracy and precision. The studied drugs include; Albuterol (ALB); a bronchodilator, Erdosteine (ERD); a mucolytic, Paracetamol (PAR); an antipyretic/analgesic, Amoxicillin (AMX); an antibiotic, Chlorpheniramine (CHR); an antihistaminic, and Guaifenesin (GUA); an expectorant. Moreover, two of the most common preservatives that are included in drug formulations used in the treatment of URTDs; Methyl Paraben (MPB) and Propyl Paraben (PPB). Many of the investigated drugs are frequently prescribed for the management of upper respiratory tract infections, particularly in pediatric populations where dosing accuracy is critically important. In such populations, even small deviations in drug concentration may lead to sub-therapeutic exposure or an increased risk of adverse effects, especially for drugs with relatively narrow therapeutic windows. Therefore, the development of reliable and accurate analytical methods for the simultaneous quantification of these drugs is essential to support quality control, therapeutic monitoring, and pharmaceutical formulation assessment. Moreover, accurate analytical determination plays an important role in ensuring drug safety and efficacy in vulnerable patient groups. Chemical structures of the studied analytes are illustrated in (Fig. 1).

A review of the literature revealed that various analytical methodologies have been developed for determination of the selected drugs as single components and in binary and ternary mixtures used for the treatment of common cold. Starting with the well-recognized drug; Paracetamol whose determination using spectroscopic, electrochemical and chromatographic techniques is widely covered in the literature^6–14^. Likewise, numerous analytical methodologies have been reported for the quantitation of Amoxicillin including high performance liquid chromatography (HPLC), high performance thin layer chromatography (HPTLC), spectrophotometric methods including continuous wavelet transform techniques^15–21^.

Moreover, several analytical methodologies were described for the quantitation of Erdosteine based on UV spectrophotometry^22–24^, HPLC using UV^25^ and tandem mass detection^26^, HPTLC^27^, and Ultra-High Performance Liquid Chromatography (UHPLC) using fluorescence^28^, and mass detectors^29^. Moving on to mixtures composed of Guaifenisin and Albuterol, they were quantitated utilizing HPLC including stability indicating methods^30–33^, micellar electrokinetic chromatography and gas chromatography with mass detection (GC-MS)^34^, and chemometrics-assisted spectrophotometric techniques^35,36^.

HPLC was employed in the determination of components frequently co-formulated in common cold preparations such as Guaifenisin, Chlorpheniramine, Methyl and Propyl parabens^37–41^ and Chlorpheniramine, Guaifenisin, and Albuterol^33^. Additionally, micellar liquid chromatography^41^ and HPTLC^42^ were exploited for the separation and concurrent estimation of mixtures comprising some of the cited drugs. Moreover, several methods were described for the multi-analyte determination of common cold preparations including the use of continuous wavelet transform spectrophotometry^43^, stability indicating ultra-performance liquid chromatographic (UPLC)^44^ and gas chromatography (GC)^45^.

Nowadays, there is a burgeoning demand for simple and sensitive analytical techniques that can detect and quantify a wide range of different analytes in a single measurement. Moreover, the establishment of comprehensive, multi-analyte analytical methodologies capable of separating and quantitating multiple analytes that are similar in their structure or pharmacological action concurrently is growing in importance in the area of Green Analytical Chemistry (GAC). The application of multi-analyte methods coincides with several principles of GAC; starting with principle **2**; reduction of sample size and number, principle **7**; minimal generation of harmful organic waste, principle **8**; use of multi-analyte methods, and principle **9**; energy and cost reduction^46^. Hence, the implementation of multi analyte methods is privileged in regular analysis in quality control laboratories as the analysis of multiple analytes in a single run conserves time, energy and plenty of other resources^47^. When compared to single component analyses, the implementation of multi-analyte techniques in pharmaceutical analysis significantly improves efficiency by allowing the simultaneous analysis of multiple compounds from a single or different samples, which minimizes expenses, time, and waste^48–55^.

The current study was driven by the absence of comprehensive analytical methods describing the concurrent determination of all the investigated drugs within a single run in the literature. The studied drugs are commonly co-administered for the treatment of URTDs, and the establishment of a versatile, broad-spectrum analytical method capable of accurately quantitating the aforementioned compounds is crucial for quality control purposes. Herein, we present a novel, straightforward, sensitive and environmentally benign HPLC-DAD method for the quantitation of Albuterol, Erdosteine, Paracetamol, Amoxicillin, Chlorpheniramine, Guaifenisin, Methyl and Propyl parabens in bulk and their pharmaceutical formulations. The proposed method was successfully verified in compliance with the International Council for Harmonization (ICH) guidelines with good overall performance. Furthermore, a thorough analysis was conducted to demonstrate the suggested method’s greenness, blueness, violet innovation and whiteness/sustainability profiles utilizing the Analytical GREEnness metric (AGREE), Analytical eco-scale, Blue Applicability Grade Index (BAGI), Click Analytical Chemistry Index (CACI), Violet Innovation Grade Index (VIGI) and Whiteness (RGB-12) assessment metrics.

## Experimental

### Instrumentation

The HPLC-DAD system comprised of Agilent 1260 series (Agilent Technologies, Santa Clara, CA, USA) (auto-injector, quaternary pump, vacuum degasser and diode array and multiple wavelength detector) connected to a computer running the Open Lab CDS ChemStation Edition C.01.09 (Agilent Technologies, Santa Clara, CA, USA). The buffer pH was monitored using a Jenway 3510 pH meter.

### Materials and reagents

**Pure sample of Erdosteine** was generously provided by Borg Pharmaceutical Industries (Alexandria, Egypt). Additionally, **Amoxicillin**,** Paracetamol**,** Albuterol**,** Guaifenisin**,** Chlorpheniramine (as maleate salt)**,** Methyl and Propyl parabens** were generously provided by Pharco Pharmaceuticals Co. (Alexandria, Egypt). HPLC-grade methanol (Sigma–Aldrich Chemie GmbH, Buchs, Switzerland), analytical grade of ortho-phosphoric acid, sodium hydroxide, potassium dihydrogen orthophosphate and ultra-purified distilled water were utilized.

Pharmaceutical formulations that were investigated include **Mucotec**^®^ capsules labelled to contain 300 mg Erdosteine (Global Nabi Pharmaceuticals, Egypt), **Anallerge**^®^ tablets containing 4 mg Chlorpheniramine (Kahira Pharmaceuticals and Chemical Industries, Egypt), **Ibiamox**^®^ capsules containing 500 mg Amoxicillin (Amoun Pharmaceutical Company, Egypt), **Adol**^®^ syrup containing Paracetamol 120 mg/5mL (Gulf Pharmaceutical Industries, U.A.E), **Dexaphen**^®^ syrup containing 2 mg/5mL CHR, Dexamethasone 0.5 mg/5mL and Methyl and Propyl parabens as preservatives (Pharco Pharmaceuticals, Alexandria, Egypt) and **Ventolin**^®^ expectorant syrup containing 1 mg/5mL Albuterol and 50 mg/5mL Guaifenisin (GlaxoSmithKline S.A.E, Cairo, Egypt). The assayed formulations were acquired from a local pharmacy.

### General procedure

#### Chromatographic system

An Inertsil ODS-3 (4.6 × 250 mm, 5 μm) column (GL Sciences, Japan) was used to complete the separation. The mobile phase system consisted of 0.025 M potassium dihydrogen orthophosphate buffer (KH_2_PO_4_) adjusted at pH 4, and methanol was gradiently eluted using an injection volume of 10 µL in the ratios and flow rates listed in (Table 1). Upon completion of each chromatographic run, the mobile phase composition was restored to the initial conditions (62% phosphate buffer and 38% methanol), and the column was allowed to equilibrate prior to the subsequent injection to maintain baseline stability and consistent retention times during continuous analyses. Chromatograms were recorded at 225 nm for Albuterol and Chlorpheniramine, 230 nm for Guaifenisin and Amoxicillin, 236 nm for Erdosteine, 250 nm for Paracetamol, and 256 nm for Methyl and Propyl parabens. The diode array detector tracked the eluent from 190 to 400 nm. Chromatographic separations were conducted at 25 °C.

**Table 1:** Gradient elution of the mobile phase applied in the proposed HPLC-DAD method.

Time (min)	KH_2_PO_4_ Buffer (%)	Methanol (%)	Flow rate (mL/min)
0	62	38	0.8
2	62	38	0.8
3	74	26	0.8
6	74	26	0.8
10	35	65	1.5
20	35	65	1.5

#### Standard solutions

Stock solutions (1 mg/mL) of the compounds under analysis were prepared by accurately weighing 10 mg of each drug into separate 10-mL volumetric flasks and completing to the volume with methanol. Every solution was kept in a refrigerator and secured against light. To obtain the mixed standard solution, appropriate aliquots of each individual stock solution were accurately transferred into a volumetric flask and diluted to the mark with methanol to produce a multi-component standard mixture containing all analytes at the desired working concentrations covering the following ranges; 1–100 µg/mL for Paracetamol, Albuterol, Chlorpheniramine, Guaifenisin, Methyl and Propyl parabens, 5–100 µg/mL for Erdosteine, and 50–500 µg/mL for Amoxicillin. The chromatographic conditions listed above were used to inject samples in triplicate for each solution. The calibration plots were produced by plotting the peak areas against the relevant concentrations.

#### Analysis of dosage forms

Separately, the contents of ten units of Mucotec^®^, Ibiamox^®^ Capsules were emptied. On the other hand, ten units of Anallerge^®^ Tablets were weighed and thoroughly crushed. In separate flasks, 15 mL of methanol was mixed with a specific quantity of each powder equivalent to 25 mg Erdosteine (Mucotec^®^), 25 mg Amoxicillin (Ibiamox*®*), and 25 mg Chlorpheniramine (Anallerge*®*). Solutions were sonicated for 20 min followed by filtration into 25 mL volumetric flasks. The remainder was washed with methanol and the final filtrate was diluted to the final volume with methanol to achieve a final concentration of 1000 µg/mL of each of Erdosteine, Amoxicillin, and Chlorpheniramine. As for the syrups, accurate proportions of 0.5 mL of Adol^®^, Dexaphen^®^, and Ventolin^®^ Syrups were added to distinct flasks and completed to the mark using methanol. The samples were further diluted to accomplish a final concentration of 100 µg/mL for Paracetamol (Adol^®^), 100 µg/mL for Chlorpheniramine (Dexaphen^®^), and 2 µg/mL of albuterol and 100 µg/mL of Guaifenisin (Ventolin^®^). Proportions of the prepared standard solutions were mixed with methanol to achieve final concentrations falling within the stated limits then analyzed as described in the previous sections. Finally, the recovered concentrations were computed from the respective calibration plots. Furthermore, each formulation was analyzed using the standard addition method to ensure accuracy in quantification within dosage forms. Accurate volumes of Albuterol, Erdosteine, Amoxicillin, Paracetamol, Guaifenisin, Chlorpheniramine, Methyl and Propyl parabens standard solutions were added to the respective sample solutions to achieve cumulative concentrations within the established linearity ranges and then examined as explained earlier. By relating the analyte response with the elevated response observed following the standard addition, recovery percentages were computed.

## Results and discussion

### Optimization of chromatographic system

The proposed HPLC-DAD approach was implemented to efficiently separate and analyze an octa-mixture of Albuterol, Erdosteine, Paracetamol, Amoxicillin, Guaifenisin, Chlorpheniramine, Methyl and Propyl Parabens in a single injection within a reasonable run time. Several trials were conducted to ensure optimal selection of the chromatographic system to guarantee appropriate resolution of the investigated compounds with adequate peak symmetry and within suitable analysis time.

Considering the stationary phase, different sorts of reversed phase C8 and C18 columns were attentively investigated. Insufficient separation among the different peaks, particularly the structurally correlated analytes Methyl and Propyl parabens, and significant peak asymmetry for other analytes was exhibited by C8 column. Hence, the Inertsil ODS-3 C18 (4.6 × 250 mm, 5 μm) was eventually selected as it produced clear separation of the eight components in a brief run time with capacity factor (K`) values ranging from 2.62 to 18.3 (Table S1 in Supplementary Material).

Furthermore, different mobile phase combinations were assessed by varying the proportions of aqueous phases and organic modifiers. Initially, several aqueous systems were investigated, comprising acetic, formic acids, and phosphate buffer. Acetic and formic acids produced broader peaks with relatively elevated capacity factor values. The use of phosphate buffer, however, led to chromatograms with sharper peaks exhibiting greater symmetry and separation efficiency expressed as theoretical plates counts (*N* = 3054-128015) and hence higher resolution values (> 1.5). Several pH values of phosphate buffer were explored, in the range 2 to 6. At pH 2, longer retention times were noted and the system was unable to elute the parabens. Increasing the pH above 4, has led to co-elution of Paracetamol and Amoxicillin and unreasonable long retention times. On the other hand, the parabens appeared as single forked peak, and no peaks were detected for Albuterol, Erdosteine, nor Guaifenisin. Lowering phosphate buffer pH to 4 resulted in an enhanced peak resolution, shape, and lowered broadness, providing the optimum balance of resolution and symmetry.

As for the organic modifier, the examined compounds were eluted using methanol and acetonitrile in different compositions. Methanol was selected as the organic modifier after evaluating alternative solvents, including the green solvent; ethanol, as it provided superior chromatographic resolution, improved peak symmetry, and shorter analysis time for the investigated analytes. Moreover, the use of methanol relatively improves the method greenness, compared to other commonly used organic solvents in HPLC analyses such as acetonitrile.

The isocratic elution of phosphate buffer and methanol in different proportions resulted in poor analyte resolution and inconvenient analysis time. Initially, isocratic elution of the mobile phase in the ratio 50:50 flowing at 1 mL/min has led to co-elution of Erdosteine and Paracetamol peaks, and at the end of the chromatogram; the parabens; Methyl and Propyl parabens showed up in a single peak. Increasing the aqueous phase (60%) to ensure separation produced a very lengthy run time (37 min) and hence a gradient system was inevitable.

Numerous gradient elutions were attempted to optimize the chromatographic system by altering the mobile phase composition and flow rate throughout the run. In order to ensure well separation of the peaks, high proportion of the phosphate buffer (62%) was programmed at the beginning of the run and then slightly increased to 74% to make sure that the early eluting peaks of ALB, maleate salt of CHR, ERD, PAR and AMX are well separated, and finally the aqueous buffer was reduced to 35% with escalating methanol proportion to 65% and elevating the flow rate to ensure elution of the remaining peaks within reasonable analysis time is achieved. The most optimal portrayal for separation of this combination was reached using a mobile phase constituting 0.025 M phosphate buffer (pH 4) and methanol gradiently eluted and pumped at the flow rates defined in Table 1, with a linear increase from 0.8 to 1.5 mL/min to guarantee sufficient separation in a reasonable analysis time.

**Fig. 2:**
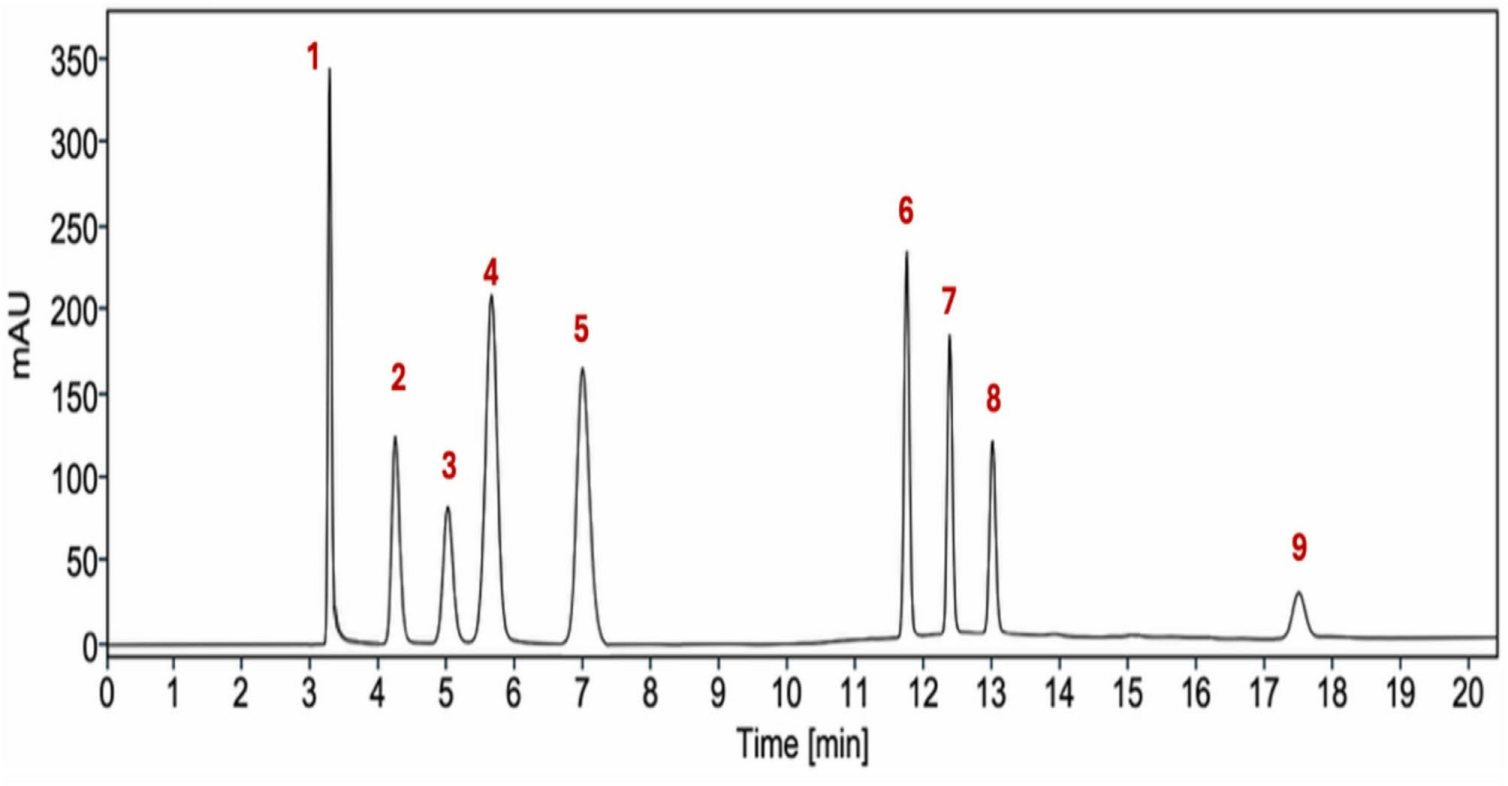
Representative HPLC chromatogram of a standard mixture of ALB (1), maleate (salt of CHR) (2), ERD (3), PAR (4), AMX (5), CHR (7), MPB (8) and PPB (9) at 230 nm. Concentration of all compounds is 100 µg/mL except AMX = 200 µg/mL.

To maximize sensitivity and yield, chromatograms were extracted at the λ_max_ of each analyte 225 nm for Albuterol and Chlorpheniramine, 236 nm for Erdosteine, 250 nm for Paracetamol, 230 nm for Amoxicillin and Guaifenisin, and 256 nm for Methyl and Propyl Parabens. Figure 2 depicts representative chromatogram for the separation of the eight compounds in less than 18 min. Initially albuterol elutes at 3.26 min followed by the first peak of Chlorpheniramine Maleate Salt (CHR-S) at 4.22 min, then the rest of the drugs namely Erdosteine, Paracetamol, Amoxicillin, Guaifenisin, Chlorpheniramine Base (CHR-B) (second peak), Methyl and Propyl parabens at 5.00, 5.64, 6.78, 11.7, 12.3, 12.9, and 17.4 respectively. Furthermore, the aforementioned chromatographic conditions resulted in an excellent separation metrics of the examined compounds. The resolution between the eluted peaks was not less than 1.65 and peaks revealed satisfying symmetry with tailing factors in the range 0.92–1.19. In addition, other system suitability characteristics were computed and confirmed to be satisfactory (Table S1 in the Supplementary File).

### Method validation

The international council for harmonization (ICH) instructions for the validation of analytical procedures were tracked in the validation and evaluation of the presented HPLC method^56^. All eight analytes showed good linearity over the concentration limits specified in Table 2 with calibration graphs illustrating high correlation coefficients (*r* ≥ 0.9991). The detector response stability is confirmed by the low percentage relative standard deviation (RSD%) of slope (< 2%) of the obtained regression equations. Likewise, the standard deviations of the intercepts were negligible, indicating method`s coherence. Furthermore, minimal dispersion of experimental data around the regression line is verified by the low significant F values. Together, these statistical measures confirm the accuracy and robustness of the method at every concentration level under investigation. Low limits of detection (LOD) and limits of quantification (LOQ), computed in accordance with ICH guidelines, revealed excellent sensitivity (Table 2). The competence of the system to detect the analytes in trace amounts with high confidence was demonstrated by the low LOD values obtained.

**Table 2:** Regression and statistical parameters for the proposed HPLC-DAD method.

Parameters/drug	ALB	ERD	PAR	AMX	GUA	CHR	MPB	PPB
Concentration range (µg/mL)	1-100	5-100	1-100	50–500	1-100	1-100	1-100	1-100
Intercept (a)	19.5	5.21	9.21	-12.8	9.33	2.72	25.4	17.61
^a^Sa	10.4	6.83	27.1	6.60	13.1	12.9	25.3	28.4
Slope (b)	19.3	15.4	52.9	14.5	10.9	14.1	42.5	37.5
^b^Sb	0.187	0.126	0.489	0.122	0.237	0.228	0.457	0.511
Sb%	0.966	0.811	0.924	0.836	2.157	1.617	1.074	1.361
Correlation coefficient (r)	0.9998	0.9999	0.9998	0.9999	0.9991	0.9996	0.9998	0.9996
^c^Sy/x	16.8	10.7	43.8	10.4	21.2	20.3	40.9	45.8
^d^F	10,718	15,196	11,716	14,312	2150	3826	8662	5394
Significance F	5.22 × 10^− 8^	2.6 × 10^− 8^	4.37 × 10^− 8^	2.93 × 10^− 8^	1.29 × 10^− 6^	9.31 × 10^− 6^	7.99 × 10^− 8^	2.06 × 10^− 7^
^e^LOD (µg/mL)	0.26	1.32	0.23	13.6	0.27	0.26	0.23	0.21
^f^LOQ (µg/mL)	0.87	4.41	0.76	45.5	0.91	0.86	0.77	0.69

^a^Standard deviation of the intercept.

^b^Standard deviation of the slope.

^c^Standard deviation of residuals.

^d^Variance ratio, equals the mean of squares due to regression divided by the mean of squares about regression (due to residuals).

^e^Limit of detection.

^f^Limit of quantification.

Accuracy was investigated in triplicate at three concentration levels for the eight analytes, and the method showed excellent recovery, with acceptable percentage relative error (Er%) (< 2%), and recovered (found) concentrations of the investigated drugs (Table 3). On the other hand, Intra and interday precision were assessed at three concentration levels producing RSD% values well below 2%, validating the method’s precision, as shown in (Table 3).

**Table 3:** Intraday and interday precision and accuracy for the determination of the selected drugs using the proposed HPLC-DAD method.

Drug		Nominal concentration (µg/mL)	Found Concentration ± SD^a^ (µg/mL)	RSD%^b^	Er%^c^
ALB	Intraday	5	4.96 ± 0.07	1.44	-0.80
		20	20.1 ± 0.28	1.41	0.36
		75	76.2 ± 0.10	0.13	1.60
	Interday	5	4.95 ± 0.02	0.45	-1.06
		20	19.7 ± 0.32	1.61	-1.37
		75	74.7 ± 1.37	1.83	-0.41
ERD	Intraday	5	4.99 ± 0.03	0.58	-0.12
		20	20.0 ± 0.17	0.85	0.17
		75	75.3 ± 0.02	0.03	0.44
	Interday	5	4.95 ± 0.06	1.19	-0.98
		20	20.1 ± 0.30	1.50	0.51
		75	73.8 ± 1.45	1.97	-1.55
PAR	Intraday	5	5.11 ± 0.02	0.43	1.13
		20	19.8 ± 0.16	0.80	-0.83
		75	75.4 ± 0.04	0.06	0.59
	Interday	5	5.06 ± 0.09	1.79	1.24
		20	20.0 ± 0.38	1.87	0.14
		75	74.7 ± 0.77	1.04	-0.35
AMX	Intraday	100	101.9 ± 0.53	0.52	1.89
		200	203.3 ± 1.54	0.76	1.66
		400	401.9 ± 0.41	0.10	0.50
	Interday	100	100.9 ± 1.06	1.05	0.85
		200	201.6 ± 2.41	1.19	0.80
		400	400.6 ± 2.51	0.63	0.15
CHR	Intraday	5	5.06 ± 0.08	1.52	1.23
		20	19.7 ± 0.15	0.76	-1.56
		75	76.4 ± 0.51	0.67	1.88
	Interday	5	5.03 ± 0.07	1.45	0.58
		20	19.8 ± 0.11	0.55	-0.94
		75	75.6 ± 0.99	1.31	0.73
GUA	Intraday	5	5.00 ± 0.03	0.67	0.11
		20	20.3 ± 0.33	1.64	1.70
		75	74.9 ± 0.17	0.22	-0.07
	Interday	5	5.03 ± 0.06	1.10	0.66
		20	19.9 ± 0.37	1.84	-0.17
		75	74.3 ± 0.96	1.30	-0.97
MPB	Intraday	5	4.94 ± 0.02	0.50	-1.26
		20	19.8 ± 0.29	1.48	-1.01
		75	75.6 ± 0.01	0.02	0.75
	Interday	5	4.91 ± 0.04	0.83	-1.73
		20	19.8 ± 0.02	0.13	-1.15
		75	75.0 ± 1.22	1.63	0.05
PPB	Intraday	5	5.0 ± 10.06	1.14	0.23
		20	19.7 ± 0.07	0.37	-1.33
		75	75.37 ± 0.09	0.12	0.50
	Interday	5	4.97 ± 0.07	1.34	-0.70
		20	19.6 ± 0.12	0.60	-1.98
		75	74.5 ± 0.84	1.13	-0.56

^a^SD = standard deviation, ^b^RSD = relative standard deviation, ^c^Er% = percentage relative error.

ALB: Albuterol; AMX: Amoxicillin; CHR: Chlorpheniramine; ERD: Erdosteine; PAR: Paracetamol; GUA: Guaiphenesin; MPB: Methyl paraben; PPB: Propyl paraben.

The capacity of the chromatographic system to selectively resolve and identify the analytes in the presence of one another and in different ratios served as confirmation of its specificity. Chromatograms for separation of these mixtures are demonstrated in Figure S1 in the supplementary file. The analysis results of laboratory-prepared mixtures for the eight analytes comprising the percentage recovery and percentage relative error (Er%) values as displayed in Table S2 in the supplementary file, were satisfactory, validating the method’s specificity and accuracy over a broad range of concentration ratios. The specificity of the suggested method was clearly verified through the analysis of pharmaceutical formulations. The absence of co-eluted extra peaks from any of the inactive components in the dosage forms was confirmed by the peak purity plots obtained using DAD (Figure S2 in the supplementary file).

Moreover, the robustness of the method was thoroughly examined by purposefully changing different critical chromatographic parameters; methanol percentage (± 2%), flow rate (± 0.1 mL/min), phosphate buffer pH (± 0.2) and detecting wavelength (± 2 nm). The results displayed in Table S3 (supplementary file) revealed that the experimented alterations did not have any significant consequence on the recorded peak areas of the investigated compounds where the calculated percentage recoveries varied between 98.6 and 101.6% and resolution among the separated peaks did not fall below 1.5.

The stability of sample solutions and standard working solutions in methanol was assessed for 24 h at room temperature and no chromatographic deviations were detected. Besides, stock solutions dissolved in methanol have demonstrated good stability for not less than two weeks once maintained at 4 °C. There was no noticeable degradation during these durations, and the retention times and peak areas of the analyzed compounds stayed constant.

### Quantitation of pharmaceutical formulations

**Fig. 3:**
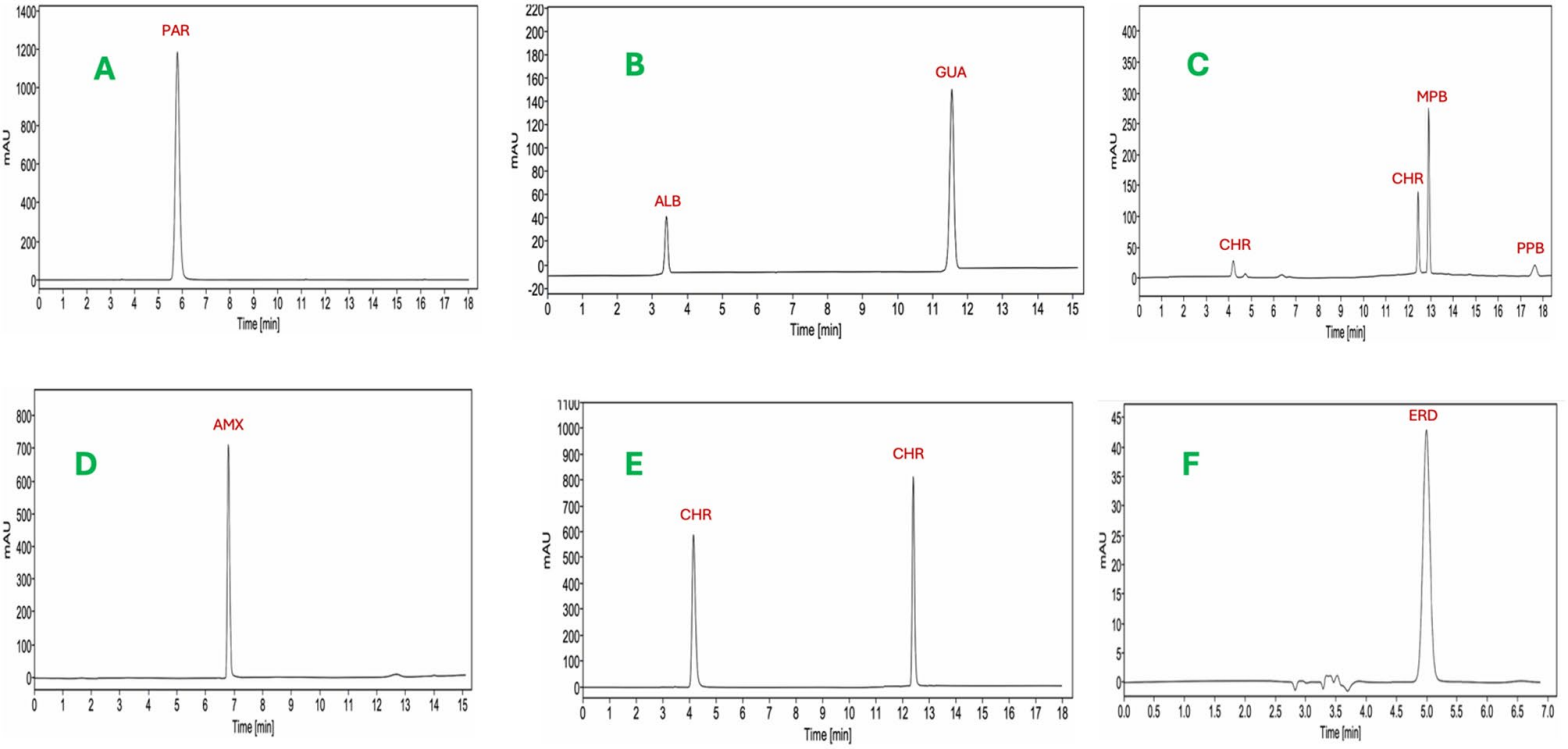
Chromatograms obtained by application of the proposed HPLC method in the determination of various common cold preparations including (**A**) Adol^®^ Syrup, (**B**) Ventolin^®^ Syrup, (**C**) Dexaphen^®^ Syrup, (**D**) Ibiamox^®^ Capsules, (**E**) Anallerge^®^ Tablets, and (**F**) Mucotec^®^ Capsules.

A wide variety of pharmaceutical formulations was used to assess the capability of the developed method to quantify the studied drugs in their pharmaceutical preparations. These include Mucotec^®^ Capsules, Ibiamox^®^ Capsules, Anallerge^®^ Tablets, Adol^®^, Ventolin^®^, and Dexaphen^®^ Syrups. Active ingredients were extracted using HPLC-grade methanol, the same solvent employed for standard stock solution preparation and subsequently diluted to achieve concentrations within the validated concentration ranges. Each compound was detected at its characteristic retention time, with no interference from excipients. Diode-array detection enabled verification of peak purity, confirming the absence of co-elution with inactive substances (Figure S2 in the supplementary file). Quantitative accuracy was assessed using the external standard and standard addition methods, and the obtained results demonstrated high recoveries along with acceptable standard deviation (SD) and relative standard deviation (RSD%) values, as presented in Table 4. These findings prove the suitability of the developed method for selective, accurate and precise real-life analysis of the investigated drugs in their dosage forms. Typical chromatograms for implementation of the described procedure for assay of different dosage forms are given in Fig. 3.

**Table 4:** Application of the proposed HPLC method for the determination of the selected drugs in their respective dosage forms.

Dosage form	Active ingredients	External standard method			Standard addition method		
		%Recovery ± SD^a^	%RSD^b^	%Er^c^	%Recovery ± SD^a^	%RSD^b^	%Er ^c^
Ventolin^®^ syrup	ALB	101.2± 0.23	0.23	1.24	101.3± 0.43	0.43	1.32
	GUA	100.6 ± 1.22	1.21	0.62	98.6 ± 0.57	0.58	-1.41
Mucotec^®^ capsules	ERD	98.4 ± 0.34	0.35	-1.63	98.2 ± 0.91	0.93	-1.84
Adol^®^ Tablets	PAR	100.2± 0.22	0.22	0.24	100.2 ± 0.32	0.32	0.24
Ibiamox^®^ capsules	AMX	100.7± 0.41	0.41	0.73	98.5 ±0.86	0.87	-1.54
Anallerge^®^ tablets	CHR	100.3 ±0.05	0.05	0.34	99.4 ± 0.51	0.51	-0.62
Dexaphen^®^ syrup	CHR	101.8± 0.04	0.04	1.82	101.4 ± 0.33	0.33	1.41
	MPB	98.8 ± 0.14	0.14	-1.22	99.7± 0.49	0.49	-0.34
	PPB	100.3 ±0.45	0.45	0.31	101.8 ± 0.37	0.36	1.81

^a^Mean percentage recovery of three replicates ± standard deviation.

^b^Percentage relative standard deviation.

^c^Percentage relative error.

### Method sustainability assessment

#### Greenness assessment

Nowadays, there is a great demand for the development of green analytical techniques in the pharmaceutical sector. This is owed to the multitude of benefits they possess over other conventional quantification techniques. These include their inexpensiveness and the consumption of minimal volumes of water-based buffers instead of harmful solvents like acetonitrile and halogenated solvents which eliminates the generation of hazardous organic wastes. In order to provide a clear and objective assessment of recently established analytical tools, it is essential to examine their ecological impact. Several greenness assessment metrics have emerged in the recent years. In this work, we have implemented the Analytical Eco-Scale tool^57^ and the Analytical GREEnness metric approach (AGREE)^58^ to ensure that a comprehensive and detailed greenness appraisal is performed for the developed method.

Analytical eco-scale (AES) is a semi-quantitative tool for assessing the environmental friendliness of analytical methodologies. It is essentially dependent on the amount of chemical reagents employed, any potential health and/or safety issues, and the energy expended to implement the investigated method^57^. The analytical eco-scale is calculated by deducting the sum of the penalty points from 100. The proposed HPLC approach received high scores (up to 85) indicating its eco-friendliness, and the penalty points considered to compute the analytical eco scale are shown in Table 5.

**Table 5:** Comprehensive assessment of the greenness, blueness and novelty of the developed HPLC method using analytical eco-scale, AGREE, BAGI, VIGI and CACI tools.

Analytical eco-scale		AGREE	BAGI	VIGI	CACI
Reagents/instrument	Penalty points				
Methanol	4				
KH_2_PO_4_	2				
HPLC energy	1				
Occupational hazard	0				
Waste	8				
Total penalty points	15				
Analytical eco-scale score	85				

AGREE is another greenness measure which consists of a clock-like figure divided into 12 segments based on the twelve green analytical chemistry (GAC) pillars. Each segment in the figure mirrors to a certain GAC principle and is colored red, yellow, and green to represent how well the analytical technique complies with the GAC idea. An overall evaluation score and color are given at the middle of the AGREE diagram on a scale of 0 to 1. The numerical appraisal of the complete operation conveyed by AGREE, allows for a thorough and simplified greenness assessment. Furthermore, the output of a method comprising the number of samples tested, the frequency of hourly analytical runs, duration, retention time, and organic solvent demand are considered by AGREE^58^. The greenness power of this study is evident, as the AGREE scores for the suggested method have reached 0.8 (Table 5).

#### Blueness assessment

Blueness index is a straightforward and rapid measuring tool that investigates the sustainability of any analytical technique employing an intuitive desktop software. This method emphasizes the applicability of an analytical technique and is based on the blue notions of white analytical chemistry concepts. The Blue Applicability Grade Index (BAGI) metric tool evaluates the steps included in sample preparation and analytical determination to be two of the most focal points in concluding the blueness of a procedure. The results obtained by BAGI software are displayed as a sequential blue color scale with distinct shades of dark blue, blue, light blue, and white representing excellent, moderate, low, or failure of the investigated technique to meet the established requirements^59^. The practical value of an analytical method is concluded if the overall blueness score exceeds 60. Given the instrument’s ease of use, the handful of steps required for sample preparation, and the widespread usage of commercially available reagents, the blueness of the developed method is confirmed with a BAGI score of 82.5 (Table 5).

#### Click analytical chemistry index (CACI)

Click analytical chemistry is a recently introduced concept that highlights the practicality and usability of analytical methods while maintaining their analytical performance. The Click analytical chemistry index (CACI) is used to score analytical methods and enable comparison between different techniques. It complements the Blue Applicability Grade Index (BAGI) by evaluating the practical aspects of analytical procedures. The CACI score is based on eight parameters, including sample size, sample preparation requirements, degree of automation, instrument portability, feasibility of the employed chemicals and instruments, method applicability, analytical sensitivity, and total analysis time. The results are displayed using a color-coded pictogram that visually represents the method performance in a user-friendly format^60^. The proposed HPLC method achieved a CACI score of 83, indicating high practical applicability (Table 5).

#### Whiteness assessment

Recently, the concept of White Analytical Chemistry (WAC) has been introduced as an extension of green analytical chemistry (GAC). This comprehensive approach evaluates analytical methods through three complementary dimensions. The red dimension assesses the analytical performance of the developed method by considering validation parameters such as limit of detection (LOD), limit of quantification (LOQ), linearity, accuracy, and precision. A higher red score indicates greater analytical reliability. The green dimension evaluates the environmental sustainability of the method by examining the type and quantity of reagents and solvents used, as well as energy consumption and waste generation; higher green scores reflect better environmental compatibility. The blue dimension measures the practical applicability of the method, taking into account factors such as instrument availability, analysis time, operational cost, and ease of use. Higher blue scores indicate greater accessibility and reproducibility^61^.

This integrated evaluation framework is known as the RGB-12 model, as it combines the three primary colors—red, green, and blue—to produce the concept of analytical “whiteness.” The closer the calculated whiteness value is to 100, the closer the method approaches the ideal analytical procedure. As illustrated in Table 6, the proposed method demonstrated superior overall efficiency compared with previously reported methods which included spectrophotometric, HPTLC, UPLC and several HPLC methods. The devised method achieved a whiteness score of approximately 95.5%, whereas the reported methods exhibited scores ranging from 91.3 to 94.7% (Table 6).

**Table 6:**
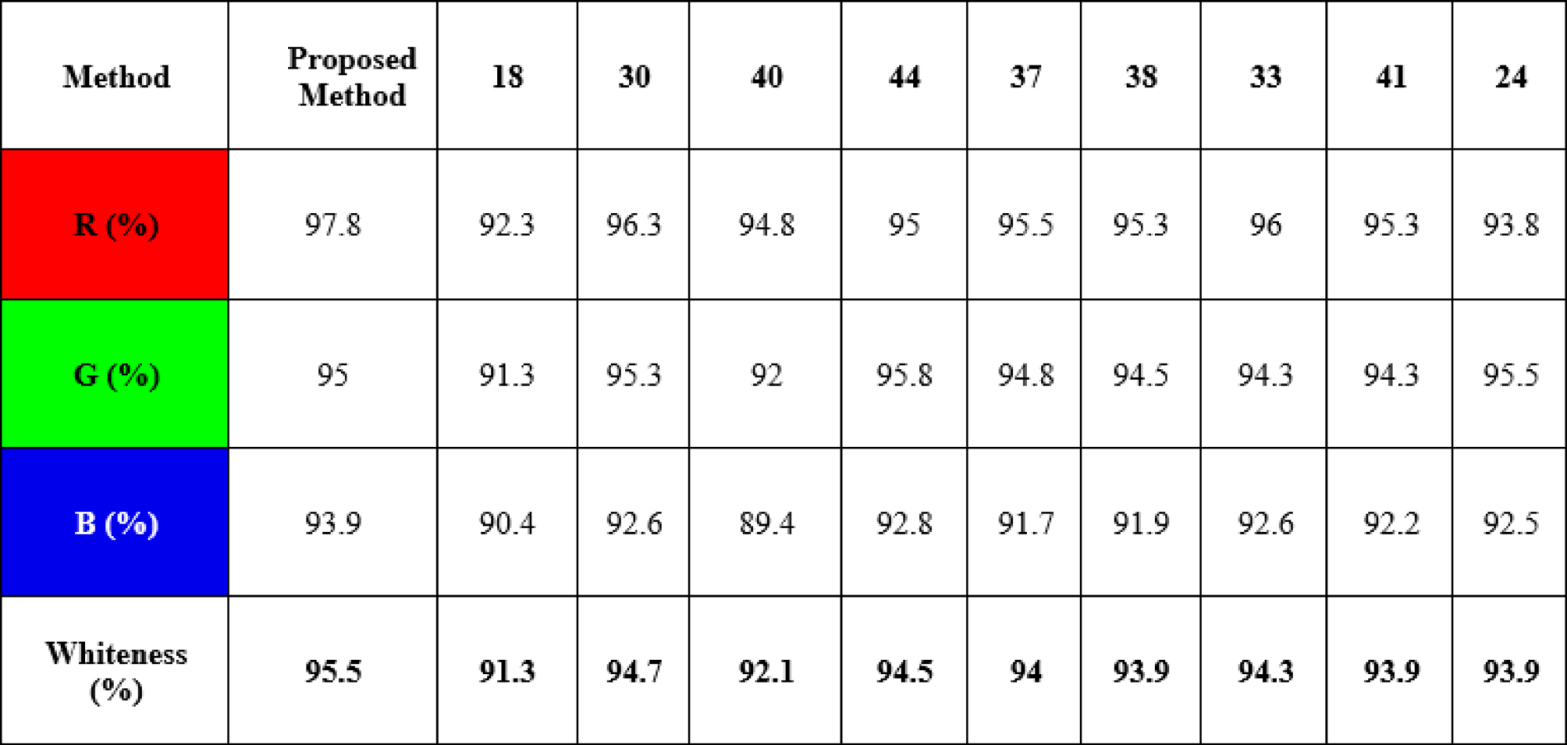
Whiteness assessment using RGB-12 algorithm of the proposed HPLC method against reported methods.

#### Violet innovation grade index (VIGI)

The violet innovation grade index (VIGI) is a recently introduced survey-driven evolution tool that evaluates the novelty extent of developed analytical methods. This evolution is based on ten innovation attributes such as the employment advanced techniques, sample preparation and preconcentration procedures which minimizes solvent consumption and provides improved sensitivity. Additionally, the assessment criteria encompass the use of sophisticated software and algorithms in data processing to cut down any human error, compliance to the fundamentals of white analytical chemistry incorporating greenness and blueness and the implementation of available tools including AGREE, GAPI and BAGI to ensure that the developed method is environmentally friendly. Furthermore, the VIGI tool concentrates on the consistency of the technique with local and international standards, usage of cutting-edge technologies, robotics, and the multifaceted nature of the method. From low innovation to high innovation, each attribute is given a value of 0, 5, or 10. The total sum of all the attributes yields a score out of 100, with a score of > 50 regarded as inventive. A violet star-shaped decagon with several shades of violet serves as the visual depiction^62^. With a VIGI score of 55, this work reveals a novel multianalyte methodology for the analysis of eight investigated compounds in their raw materials and dosage forms, giving the method an advantage over previously published methods in the literature (Table 5).

## Conclusion

This work discusses a newly developed and validated HPLC-DAD method for the concurrent assay of eight compounds namely; Albuterol, Erdosteine, Paracetamol, Amoxicillin, Chlorpheniramine, and Guaifenesin, and two of the most common additives that are incorporated in URTDs medications; Methyl (MPB) and Propyl Parabens (PPB)) in their raw materials and pharmaceutical formulations. To our present knowledge, no similar report in the literature was found capable of resolving and quantitating this octa-mixture in a single run within a reasonable analysis time, hence allowing for the quantitation of a variety of pharmaceutical formulations under identical operational settings. The described method was validated and effectively implemented in the estimation of the eight stated compounds in bulk, synthetic mixtures and in pharmaceutical formulations where satisfactory results were attained. Applying the greenness (AES and AGREE), blueness (BAGI), Whiteness (RGB-12 model), Click Analytical Chemistry Index (CACI) and Violet Innovation Grade Index (VIGI) assessment metrics verified that the suggested approach offers an acceptable compromise between economic, practicality, ecological and analytical perspectives. Lastly, quality control laboratories would benefit greatly from the development of such multianalyte, adaptable procedures in terms of economy, rapidity, sensitivity, and sustainability.

## Supplementary Information

Below is the link to the electronic supplementary material.

Supplementary Material 1

## Data Availability

All of the data analyzed in this study is included in the manuscript and the supplementary file.
