# Supplementary material for: Sustainable multifaceted HPLC approach for concurrent quantitation of an octa-mixture used in upper respiratory therapy with a five-dimensional sustainability assessment
Source: Sci Rep. 2026 Apr 13;16:12240. doi: 10.1038/s41598-026-45971-7 (PMC13076651; doi:10.1038/s41598-026-45971-7)
Supplement: Supplementary file 1 — Supplementary Material 1 [file 41598_2026_45971_MOESM1_ESM.docx]

**Sustainable Multifaceted HPLC Approach for Concurrent Quantitation of an Octa-mixture Used in Upper Respiratory Therapy with a Five-dimensional Sustainability Assessment**

**Supplementary Material**

**Table S1. System suitability parameters for the proposed HPLC-DAD method**

| **Analyte** | **t_R_**  **(min)** | **Capacity Factor**  **(K`)** | **Selectivity**  **(α)** | **Resolution**  **(R_s_)** | **Tailing**  **Factor (T)** | **Number of Theoretical Plates**  **(N)** |
| --- | --- | --- | --- | --- | --- | --- |
| **ALB** | 3.26 | 2.62 | - | - | 1.13 | 3054 |
| **CHR-S** | 4.22 | 9.55 | 1.336 | 2.97 | 1.19 | 6075 |
| **ERD** | 5.00 | 4.56 | 1.204 | 3.18 | 1.08 | 4475 |
| **PAR** | 5.64 | 5.27 | 1.139 | 2.14 | 0.99 | 3942 |
| **AMX** | 6.78 | 6.53 | 1.218 | 1.65 | 1.15 | 6278 |
| **GUA** | 11.7 | 12.01 | 2.162 | 26.2 | 0.92 | 95064 |
| **CHR-B** | 12.3 | 12.67 | 1.056 | 4.44 | 1.11 | 112744 |
| **MPB** | 12.9 | 13.33 | 1.053 | 4.10 | 1.09 | 95375 |
| **PPB** | 17.4 | 18.33 | 1.357 | 21.7 | 1.05 | 128015 |
| † Calculated as RSD% of five determinations.  ^*^ Recommended system suitability parameters: K`= 2-10, R_S_ ≥ 1.5, N > 2000, α ≥ 1.1, and T = 0.9-1.2 | | | | | | |

**Table S2: Determination of ALB, ERD, PAR, AMX, GUA, CHR, MPB, and PPB in laboratory-prepared mixtures using the proposed HPLC method.**

| **Drug** | **Synthetic Mixture 1** | | **Synthetic Mixture 2** | | **Synthetic Mixture 3** | |
| --- | --- | --- | --- | --- | --- | --- |
|  | **Nominal concentration (μg/mL)** | **% Recovery ^a^ (%E_r_)^b^** | **Nominal concentration (μg/mL)** | **% Recovery ^a^ (%E_r_)^b^** | **Nominal concentration (μg/mL)** | **% Recovery ^a^ (%E_r_)^b^** |
| **ALB** | 10 | 101.4 (1.4) | 15 | 101.8 (1.8) | 30 | 99.4 (-0.6) |
| **ERD** | 10 | 101.9 (1.9) | 15 | 100.3 (0.3) | 60 | 100.5 (0.5) |
| **PAR** | 30 | 101.2 (1.2) | 50 | 99.4 (-0.6) | 100 | 101.7 (1.7) |
| **AMX** | 200 | 99.2 (-0.8) | 250 | 100.4 (0.4) | 500 | 98.7 (-1.3) |
| **GUA** | 10 | 101.7 (1.7) | 15 | 99.6 (-0.4) | 30 | 99.9 (-0.1) |
| **CHR** | 10 | 100.6 (0.6) | 15 | 101.1 (1.1) | 30 | 101.4 (1.4) |
| **MPB** | 10 | 101.2 (1.2) | 15 | 99.5 (-0.5) | 30 | 98.5 (-1.5) |
| **PPB** | 10 | 100.4 (0.4) | 15 | 101.4 (1.4) | 30 | 98.9 (-1.1) |

^a^ Mean percentage recovery for three determinations.

^b^ Percentage relative error.

**Table (S3):** **Robustness evaluation of the proposed HPLC method for the determination of the selected drugs.**

| **Parameters** | **ALB** | **ERD** | **PAR** | **AMX** | **GUA** | **CHR** | **MPB** | **PPB** |
| --- | --- | --- | --- | --- | --- | --- | --- | --- |
|  | **Recovery % ± SD^a^** | **Recovery % ± SD^a^** | **Recovery % ± SD^a^** | **Recovery % ± SD^a^** | **Recovery % ± SD^a^** | **Recovery % ± SD^a^** | **Recovery % ± SD^a^** | **Recovery % ± SD^a^** |
| **Buffer pH + 0.2** | 100.1 ± 0.26 | 99.8±0.24 | 100.2±0.25 | 100.8±0.28 | 100.4±0.15 | 100.2±0.14 | 99.8±0.16 | 101.2±0.22 |
| **Buffer pH - 0.2** | 100.2 ± 0.28 | 98.7±0.37 | 100.5±0.31 | 98.7±0.36 | 100.8±0.27 | 100.5±0.19 | 98.7±0.25 | 101.1±0.33 |
| **Methanol ratio + 2%** | 101.6 ± 0.36 | 100.3±0.29 | 100.2±0.46 | 99.9±0.31 | 101.3±0.15 | 100.9±0.23 | 100.3±0.42 | 98.9±0.14 |
| **Methanol ratio - 2%** | 100.8 ± 0.47 | 101.3±0.34 | 100.8±0.43 | 100.4±0.16 | 101.1±0.13 | 100.1±0.45 | 100.6±0.22 | 100.3±0.26 |
| **Detection wavelength + 2 nm** | 100.9 ± 0.25 | 100.3±0.25 | 100.9±0.35 | 101.6±0.18 | 100.7±0.35 | 100.3±0.32 | 100.9±0.34 | 100.8±0.18 |
| **Detection wavelength - 2 nm** | 101.3 ± 0.39 | 100.2±0.16 | 98.7±0.22 | 100.3±0.13 | 100.3±0.26 | 100.6±0.27 | 101.2±0.26 | 100.9±0.22 |
| **Flow rate + 0.1** | 100.7 ± 0.43 | 100.6±0.19 | 99.3±0.31 | 100.6±0.26 | 100.4±0.23 | 100.2±0.31 | 100.4±0.22 | 101.2±0.32 |
| **Flow rate - 0.1** | 100.4 ± 0.29 | 100.4±0.26 | 98.6±0.27 | 100.2±0.14 | 101.3±0.31 | 100.3±0.22 | 100.7±0.24 | 100.6±0.29 |

^a^ Mean percentage recovery for three determinations **±** standard deviation

**
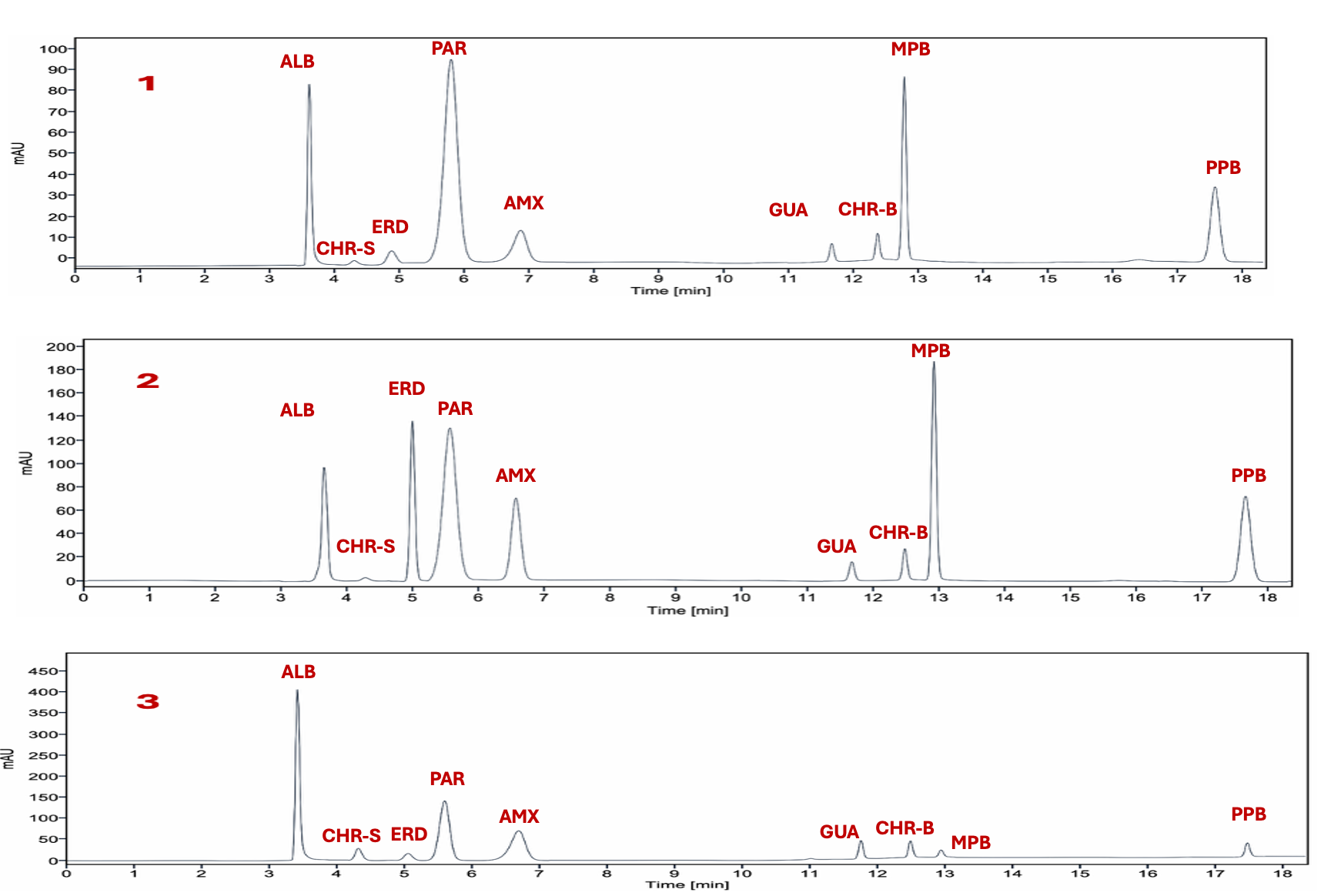
**

**Figure S1: Chromatograms for the separation of ALB, ERD, PAR, AMX, GUA, CHR, MPB, and PPB in laboratory-prepared mixtures with different concentrations (Mixture 1, 2, and 3) using the proposed HPLC method.**

**
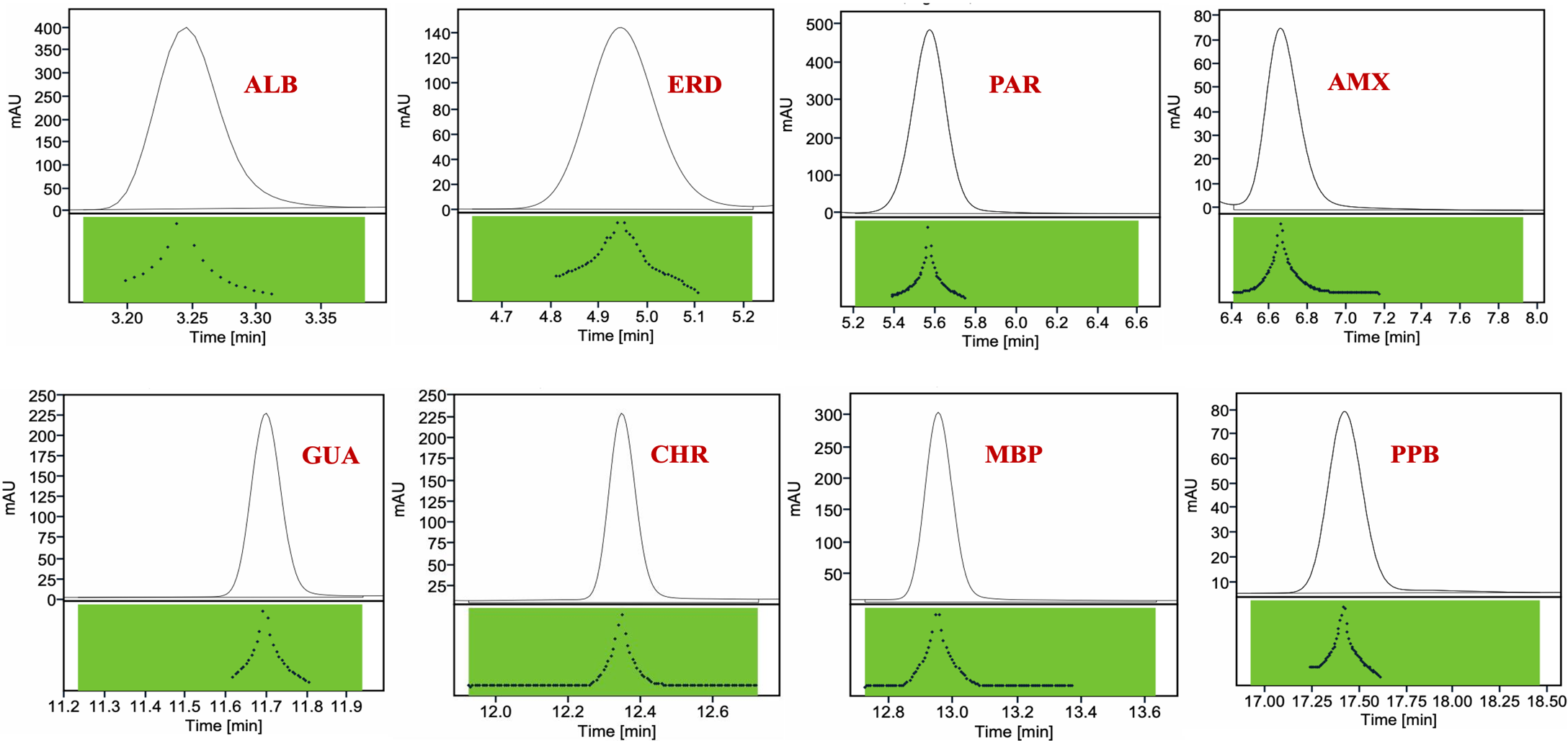
**

**Figure S2: Similarity curves illustrating peak purity of ALB, ERD, PAR, AMX, GUA, CHR, MPB, and PPB obtained using the proposed HPLC method.**

**
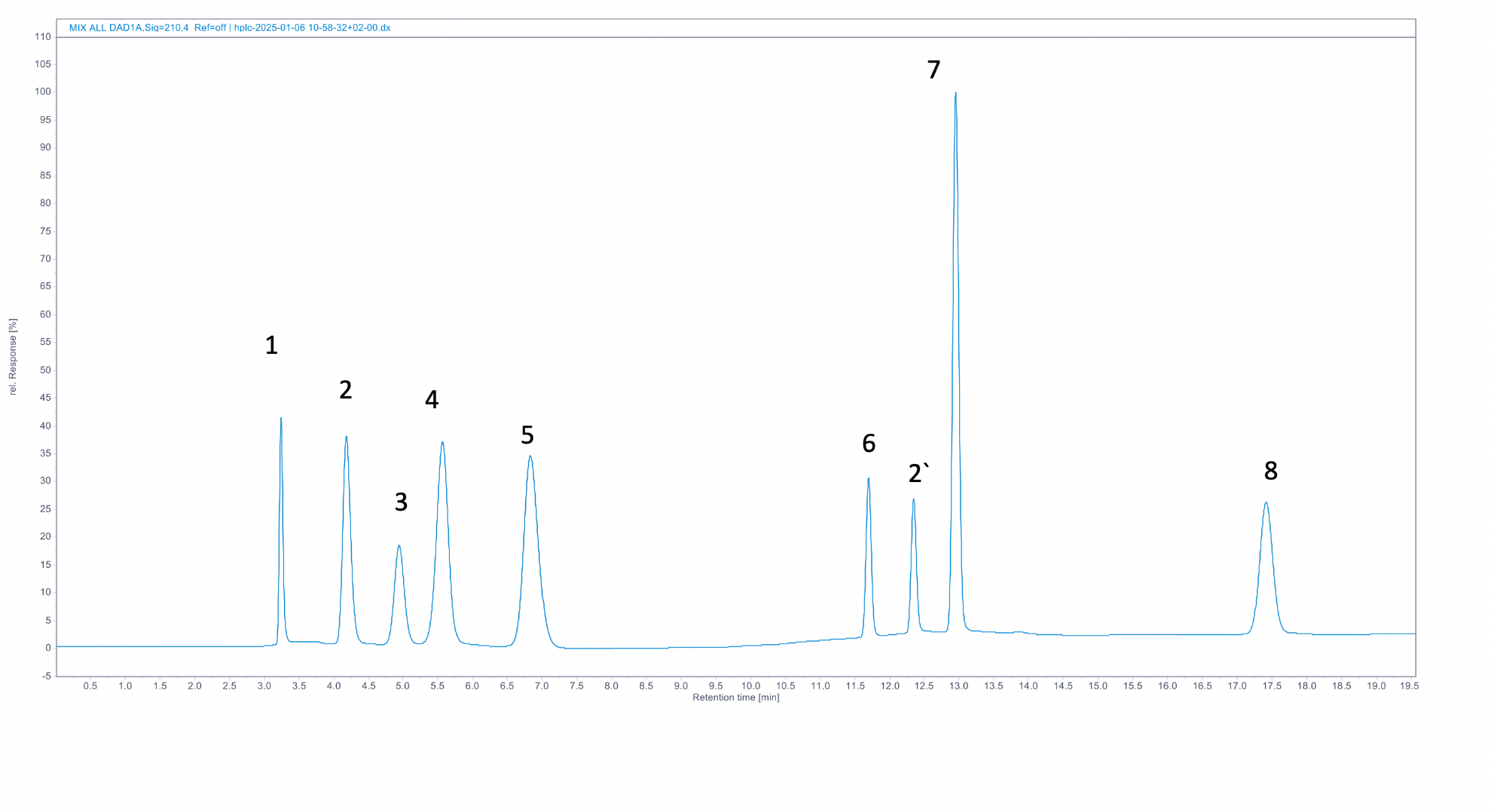
 Figure S3: Representative HPLC chromatogram of a standard mixture of ALB (1), CHR-S (2), ERD (3), PAR (4), AMX (5), GUA (6), CHR-B (7), MPB (8) and PPB (9) at 210 nm. Concentration of all compounds is 100 μg/mL except AMX = 200 μg/mL.**

**
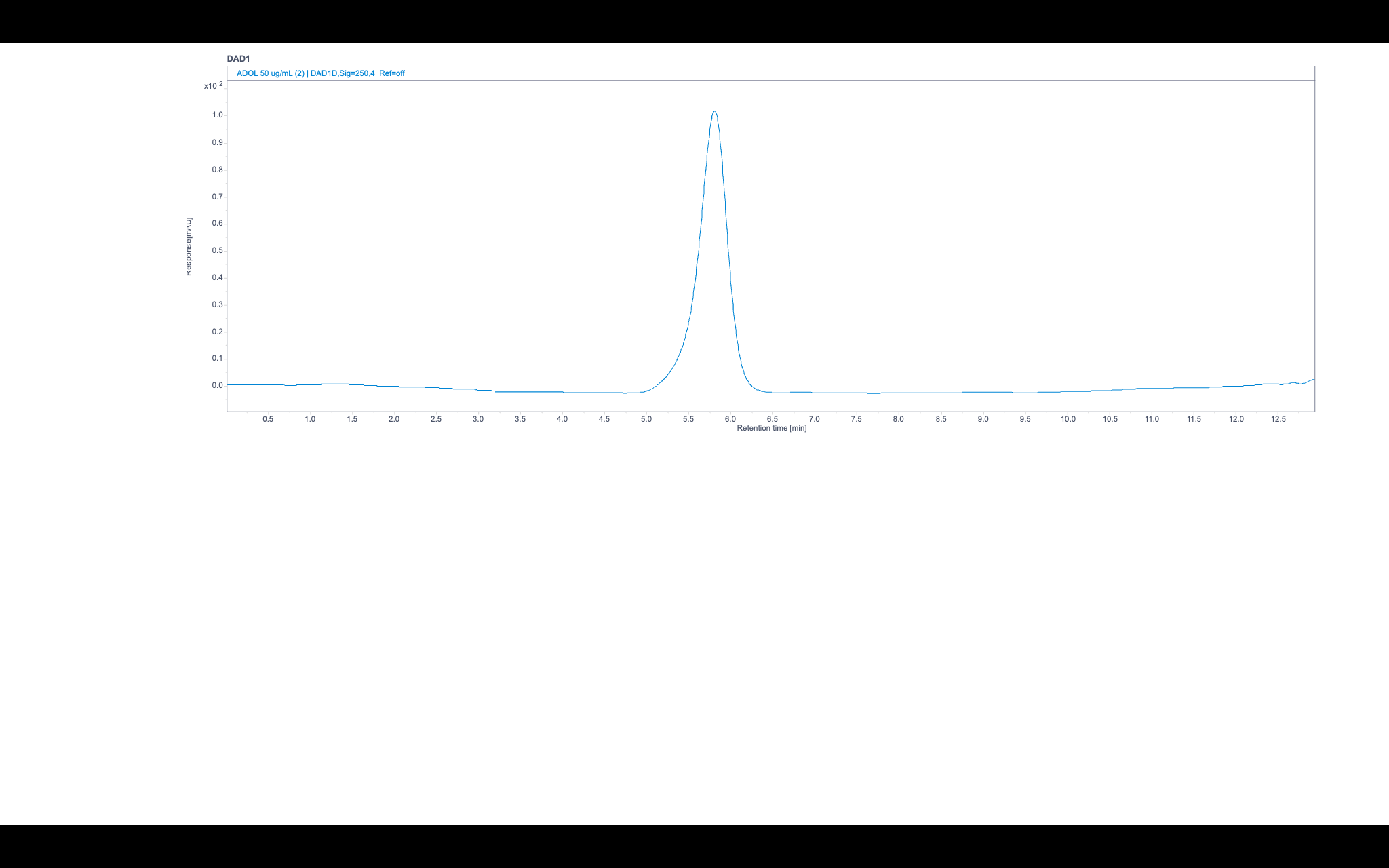
**

**(A)**

**
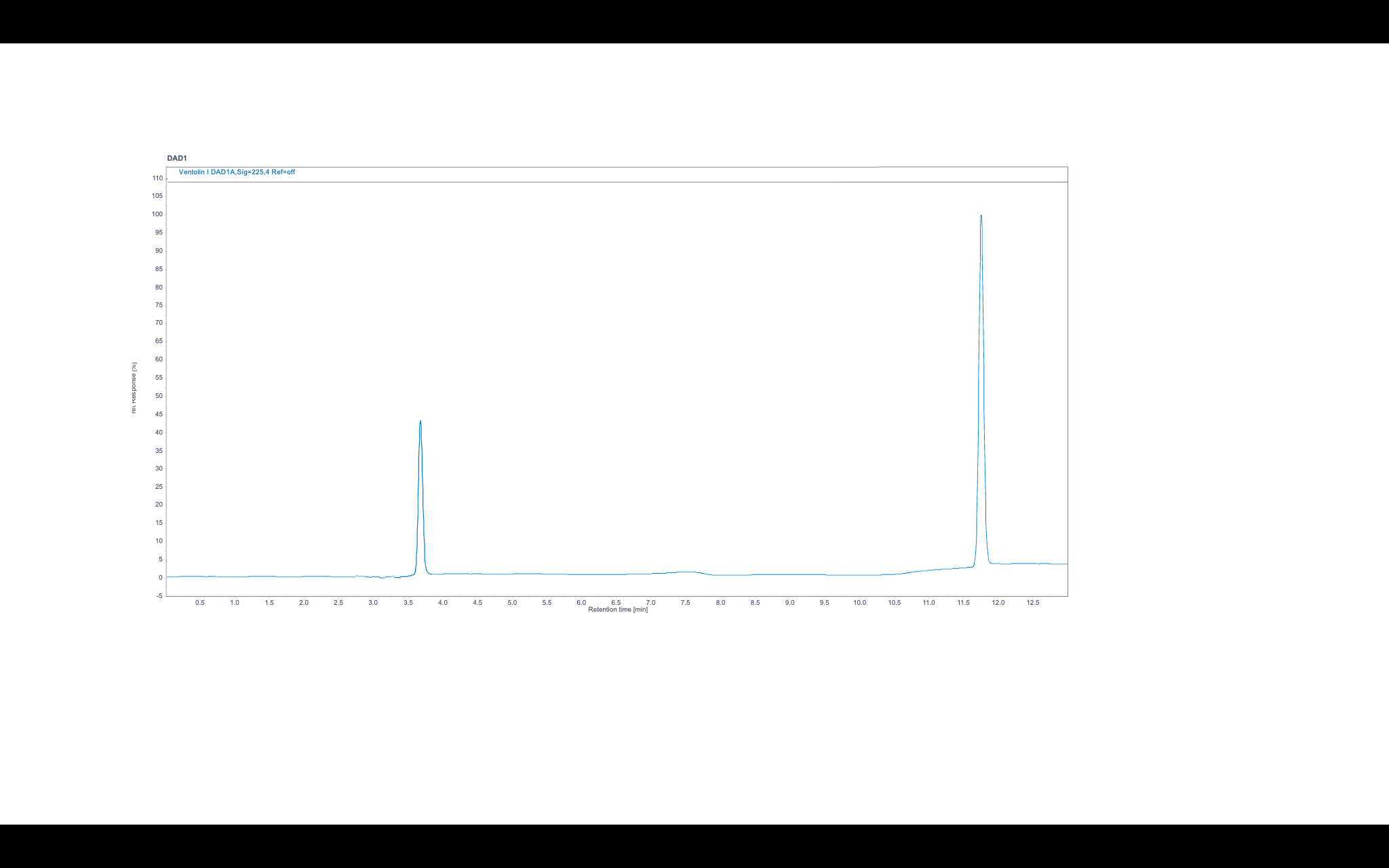
**

**(B)**

**
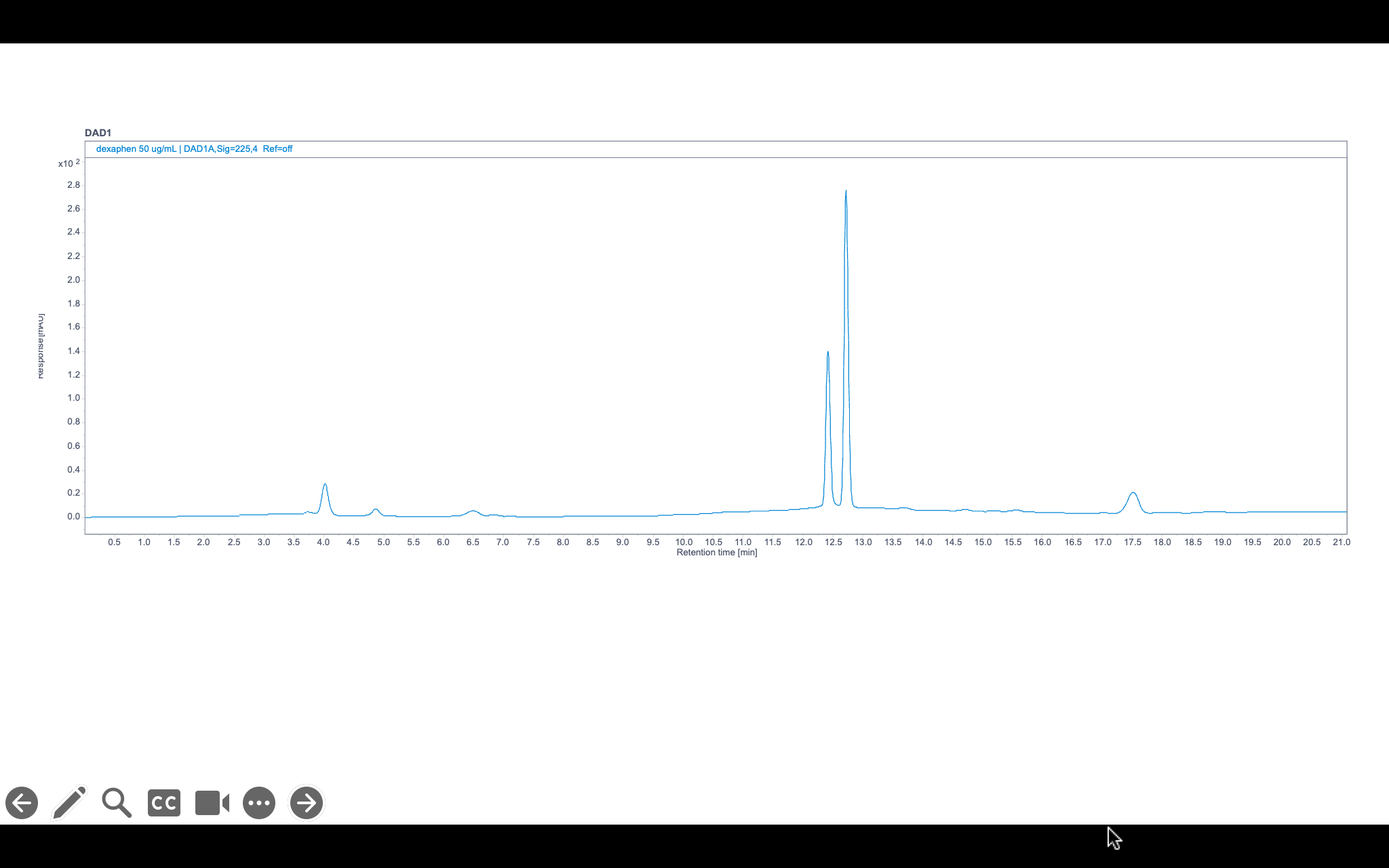
**

**(C)**

**
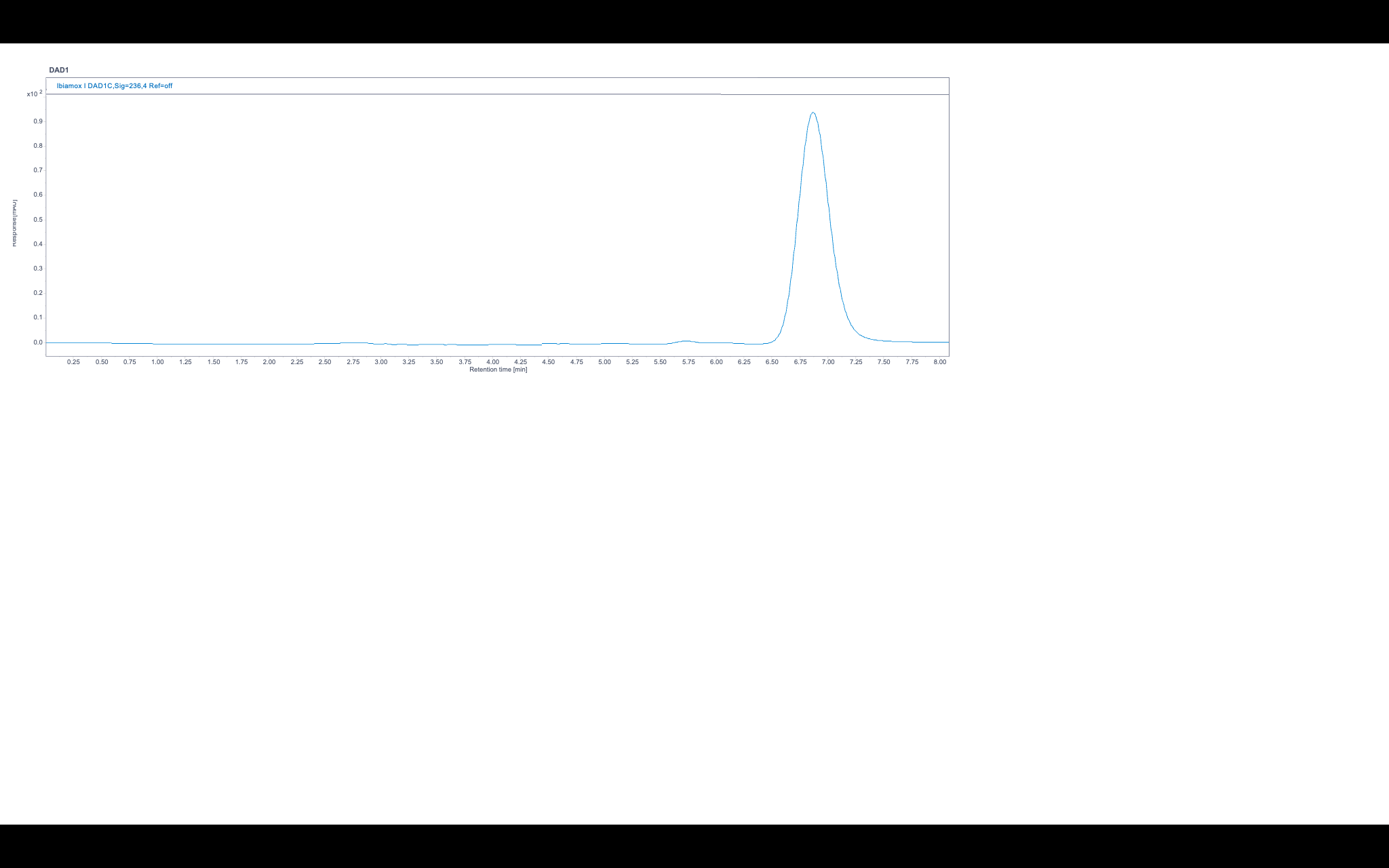
**

**(D)**

**
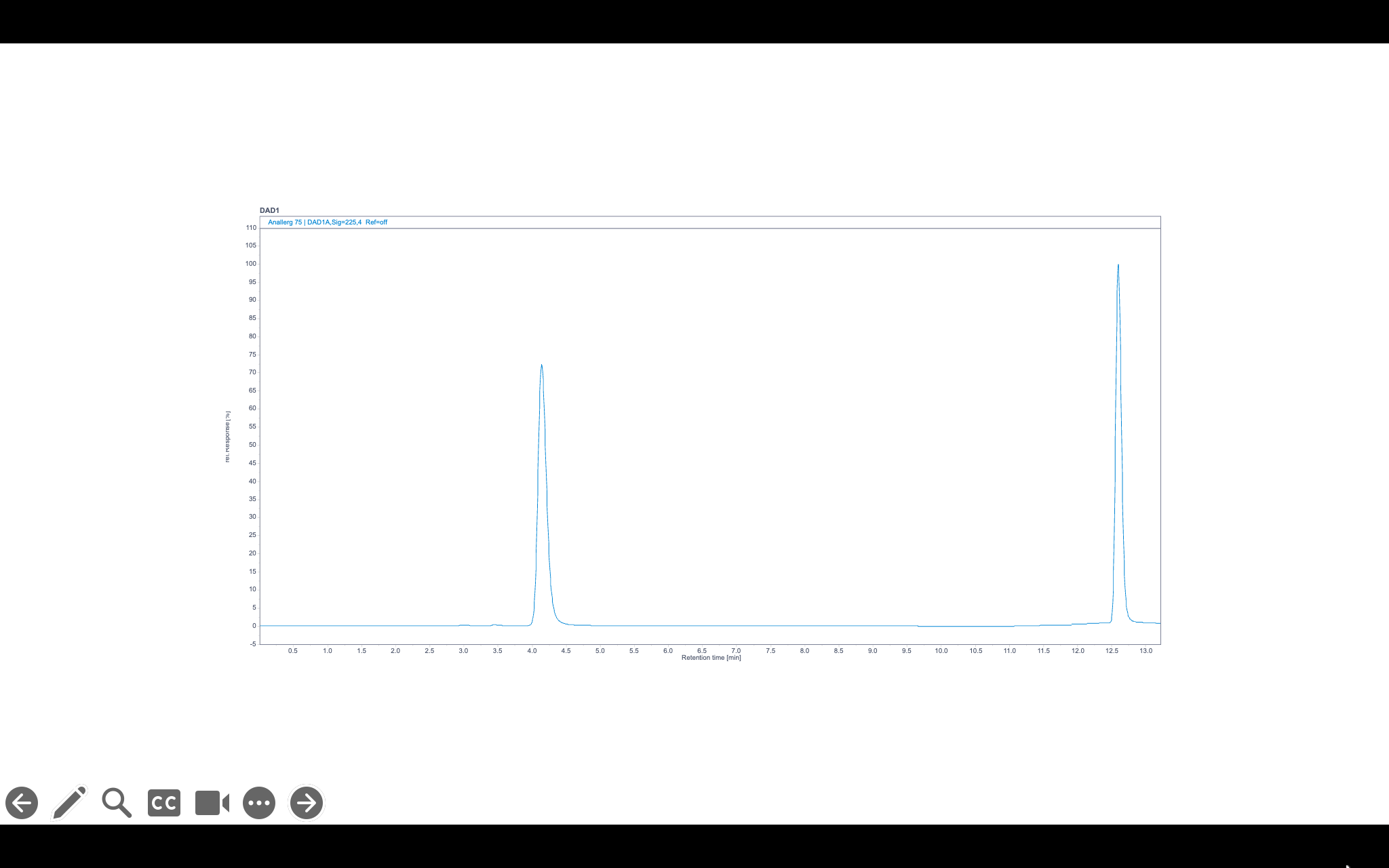
**

**(E)**

**
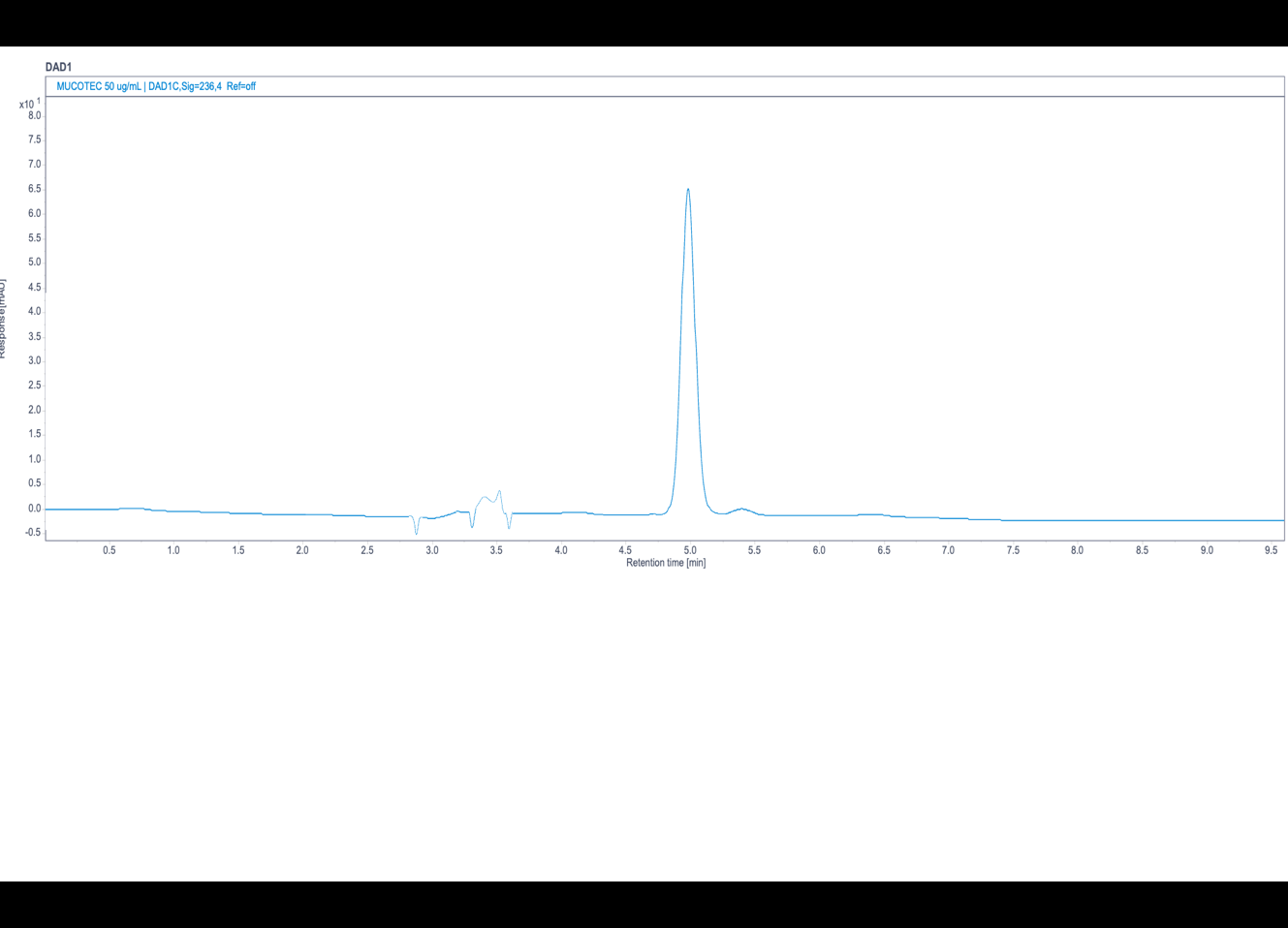
**

**(F)**

**Figure S4: Raw Chromatograms obtained by application of the proposed HPLC method in the determination of various common cold preparations including (A) Adol® Syrup, (B) Ventolin® Syrup, (C) Dexaphen® Syrup, (D) Ibiamox® Capsules, (E) Anallerge® Tablets, and (F) Mucotec® Capsules.**
